# Microstructure and Mechanical Properties of Dissimilar Friction Stir Welded T-Lap Joints Between AA5083 and AA7020

**DOI:** 10.3390/ma19153286

**Published:** 2026-08-03

**Authors:** Janusz Torzewski, Tomasz Helt, Robert Kosturek, Michal Jambor, Michal Černý, Janusz Mierzyński, Marcin Wachowski

**Affiliations:** 1 Faculty of Mechanical Engineering, Military University of Technology, gen. S. Kaliskiego 2 Str., 00-908 Warsaw, Poland; thelt@mon.gov.pl (T.H.); marcin.wachowski@wat.edu.pl (M.W.); 2 Institute of Physics of Materials, Czech Academy of Sciences, Žižkova 513/22, 61600 Brno, Czech Republic; jambor@ipm.cz (M.J.); cerny@ipm.cz (M.Č.)

**Keywords:** friction stir welding, dissimilar alloys joining, microstructure, T-joints

## Abstract

This paper investigates the microstructural evolution and mechanical performance of dissimilar friction stir-welded (FSW) T-joints produced from AA5083-H111 and AA7020-T651 aluminium alloys. The study focuses on the effects of tool rotational speed, traverse speed, and tool pin length on joint quality and mechanical strength. Welding experiments were conducted at two rotational speeds (400 and 700 rpm) and three traverse speeds (50, 100, and 200 mm/min) using two Triflute tools with pin lengths of 4.8 mm and 6.8 mm. The welded joints were examined by macro- and microstructural observations, microhardness measurements, and bending tests performed under pull-out loading conditions. The results showed that the use of the longer pin significantly improved material mixing and reduced the extent of internal defects, resulting in higher bending strength. Increasing the traverse speed improved the mechanical performance, whereas an excessively high rotational speed reduced joint strength due to increased heat input and unfavourable microstructural changes. Microhardness profiles showed no reduction in hardness within the heat-affected zones relative to the base materials and indicated localised hardening in the stir zone. For the investigated material configuration and tool geometries, the highest joint strength was obtained at a rotational speed of 400 rpm and a traverse speed of 200 mm/min using the longer-pin tool. The study confirms the critical role of process parameter optimisation and tool geometry in producing high-quality dissimilar FSW T-joints suitable for lightweight structural applications.

## 1. Introduction

There is ongoing interest in lightweight aluminium alloys across many industrial sectors, especially in the aerospace and shipbuilding industries [1,2,3]. Important properties that contribute to their popularity are corrosion resistance and high specific strength. At the same time, T-joints are commonly used in structural applications because they significantly improve structural stiffness and moment of inertia [4,5]. Thus, combining aluminium alloys in a T-joint configuration allows manufacturers to reduce structural weight and achieve significant material savings. Traditionally, T-joints are produced using welding or riveting technologies. Riveted joints are popular in aircraft structures despite adding weight and causing high stress concentrations [6]. Considering these limitations, welded joints appear to be a more suitable solution for meeting increasingly stringent requirements, as they eliminate the need for additional elements such as rivets and allow the production of watertight joints. However, welding has major limitations when joining 2xxx and 7xxx series aluminium alloys, which are generally considered unweldable. As reported in the literature [7,8,9,10], undesirable porosity formation and solidification cracking can deteriorate joint quality, making it difficult to ensure structural safety. Hence, it is necessary to introduce innovative methods for joining dissimilar materials to improve joint quality.

A significant advantage of FSW is its ability to join dissimilar materials, such as different aluminium alloys [11,12] or even aluminium and steel [13,14]. Appropriate selection and arrangement of materials can provide significant benefits in the design of complex structures. Studies of T-joints produced by friction stir welding (FSW) include a wide range of metallographic examinations and mechanical tests aimed at optimising joint quality. Research typically focuses on grain size and recrystallisation in different weld zones: the stir zone (SZ), the thermomechanically affected zone (TMAZ) and the heat-affected zone (HAZ) [15,16]. Optical microscopy (OM), scanning electron microscopy (SEM) and electron backscatter diffraction (EBSD) are commonly used for these analyses. Microstructural analysis also enables the identification of welding defects. Researchers focus on identifying and eliminating defects such as “lack of bonding,” “hooks,” “wormholes,” and “kissing bonds” [17,18,19]. Microhardness measurements are performed along the cross-sections of the skin and the stiffener [15,17], allowing the identification of material softening in the weld region and areas exhibiting significant changes in hardness. The strength of T-joints is assessed by tensile tests performed in the skin and stiffener directions. Pull-out tests are considered more representative of the actual loading conditions of T-joints because they better simulate the forces acting during the pull-out of the stiffener [20]. Researchers have also analysed other parameters associated with the friction stir welding process. In the study by Aghajani Derazkola et al., the effect of tool tilt angle (TTA) on the properties of T-joints made of Al-Mg-Si alloys was analysed [21]. Computational fluid dynamics (CFD) was used to investigate the effect of TTA on the thermal history, material flow rate, strain rate, and viscosity changes in the Al-Mg-Si alloy. The results showed that as TTA increased, higher temperatures were generated on the advancing side (AS), along with increased friction and axial forces. However, excessive stirring led to the formation of surface flash and tunnelling defects at the skin-flange interface. Jesus et al. evaluated friction stir welding of aluminium T-joints in T-butt and T-lap configurations using three different tool geometries on AA5083-H111 and AA6082-T6 plates [22]. Tunnel defects, kissing bonds, and oxide lines were observed in the welds made with a pyramidal tool, while welds made with a conical tool showed only kissing-bond defects. Welds made with a progressive tool did not reveal significant defects. No significant changes in hardness were observed for any of the investigated combinations of tool and joint geometry, and the best mechanical properties were obtained for welds made with a progressive tool in the T-butt configuration. Wagner et al. investigated single-pass and two-pass friction stir-welded T-joints produced in AA2219-T31 alloy [23]. The study focused on the mechanisms of bond failure in single-pass welds, including analyses of the microstructure, hardness, mechanical properties, and crack locations. A second welding pass was applied to the initial weld to eliminate bonding defects. The use of a second welding pass significantly improved joint performance, achieving up to 90% of the tensile strength in the skin direction and 95% in the stiffener direction for the AA2219-T31 alloy under investigation. In a study by Memon et al., the effect of tool offset (TO) on friction stir welding of an Al-Mg-Si alloy in stir and T-joint configurations was investigated [24]. The process was simulated using computational fluid dynamics (CFD) to better understand material flow, mixing, and joining between the skin and the flange during friction stir welding. According to the results, the most favourable material flow could only be achieved using an appropriate TO, which provided sufficient material to fill the weld line and resulted in the highest proportion of flange material within the stir zone (SZ). Among the cases analysed, the strongest joint was obtained with a TO of + 0.2 mm towards the advancing side (AS), retaining more than 60% of the strength of the base aluminium alloy.

The problem of using a too-short pin in the tool in the friction stir welding process for T-joints has been addressed in the literature. This can lead to several structural defects, primarily due to insufficient penetration depth and limited vertical material flow. The most frequently reported defect is a lack of bonding, which manifests as a remnant of the unwelded surface at the bottom of the weld line. This occurs when the depth of downward flow of the material exceeds the tool probe’s penetration depth into the stinger blank. As a result, the original contact surface is not broken down and mixed, creating a crevice or a weakened mechanical connection [18,22,25]. Bonding line defects are caused by using an FSW tool pin that is too short, which reduces the efficiency of vertical flow generation. As a result, the horizontal oxide layer at the original interface is not effectively fragmented and removed from the weld zone. This defect appears as continuous, dark oxide lines along the interface, drastically reducing the joint strength. Furthermore, it leads to the formation of separate horizontal flows with weak mutual interference, preventing the formation of a uniform “onion shell” structure along the entire weld height [22]. During strength tests, samples with pins that are too short tend to break directly at the corners of the T-joint, where a clear lack of pin penetration is visible [26].

Previous studies have demonstrated that strong T-joints can be successfully produced by friction stir welding (FSW) of dissimilar aluminium alloys, including alloys from the 7xxx and 5xxx series. The main goal of this work is to supplement the literature on FSW joints with studies of T-joints made of various aluminium alloys in a specific configuration, namely AA5083 and AA7020. The use of the AA5083 alloy as a skin is significant due to its exceptional corrosion resistance. The AA7020 alloy was used as a stringer due to its high mechanical strength. However, a thorough understanding of the effects of welding process parameters and tool geometry on the characteristics of these joints is still necessary. The present study investigated various FSW processing conditions and two tools with different pin lengths. Experiments were conducted at two tool rotational speeds (400 rpm and 700 rpm) and three traverse speeds (50 mm/min, 100 mm/min and 200 mm/min) using tools with pin lengths of 4.8 mm and 6.8 mm. Microfractographic examination of the joints and mechanical evaluation, including microhardness measurements and bending tests, were conducted to assess the properties of each joint type. The results showed that T-joints produced with the longer-pin tool exhibited better mechanical properties, including higher bending strength and a stable hardness distribution. Analyses of the fracture surfaces and crack paths confirmed the ability to produce dissimilar T-joints without characteristic defects that significantly reduce mechanical performance. The results confirm the potential of FSW technology for producing durable, lightweight structures for applications in the maritime industry.

## 2. Materials and Experimental Procedures

FSW experiments on T-joints were carried out using two aluminium alloys, AA5083-H111 and AA7020-T651 (Table 1). Aluminium alloy AA5083-H111 belongs to the 5xxx series, with magnesium as the principal alloying element. The H111 temper indicates that the material has undergone some strain hardening during shaping, although to a lesser extent than that required for the H11 temper. AA7020-T651 belongs to the 7xxx series, in which zinc and magnesium are the principal alloying elements, and is strengthened by precipitation hardening. The T651 temper involves solution heat treatment, followed by stress relief through controlled stretching, typically resulting in a permanent deformation of approximately 1–3%, and subsequent artificial ageing.

Table 2 summarises the mechanical properties of the tested alloys. The presented data were obtained in our previous study [27].

**Figure 1:**
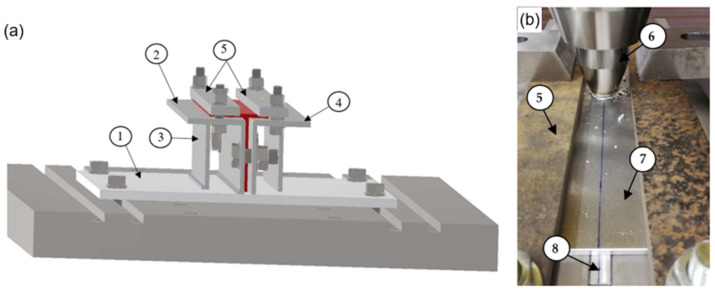
Schematic of the tooling used to produce T-joints (**a**): 1—base, 2—fixed angle, 3—stiffening flat bar, 4—movable angle, 5—clamping flat bars and technological station during the connection (**b**): 5—clamping flat bars, 6—FSW tool, 7—skin, 8—stiffener.

To produce the T-joints, special tooling was designed and manufactured at the Military University of Technology (Figure 1). The tooling enabled the specimens to be welded using an ESAB FSW Legio 4UT machine (ESAB, Goteborg, Sweden). Figure 1b shows the device during joint formation, with the FSW tool pin piercing the skin. This T-joint preparation method was adopted during the research. If the possibility of using the proposed material configuration for the construction of a ship shell is considered, the narrow space between the stringers (reinforcements) often prevents the introduction of a wide spindle and FSW welding tool from the inside.

**Figure 2:**
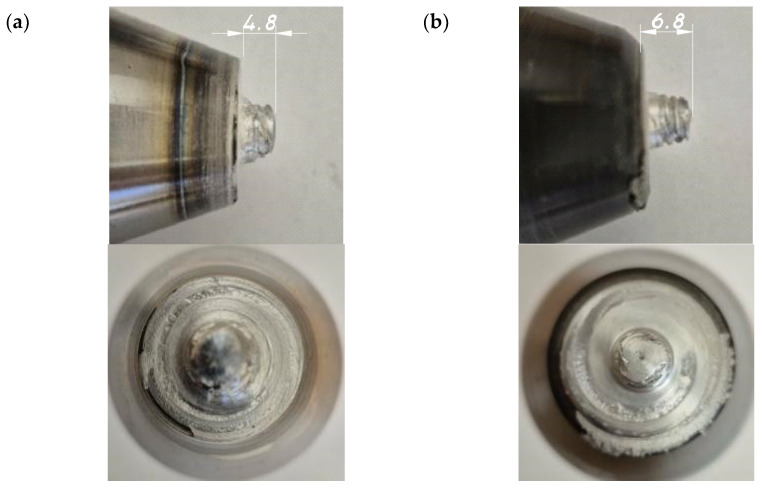
Photographs of tools used to produce T-joints: (**a**) tool 1, (**b**) tool 2.

Sheets of AA5083, with a thickness of 4 mm, and AA7020, with a thickness of 8 mm, were cut into blanks with dimensions of 300 mm × 100 mm and 300 mm × 80 mm, respectively. AA5083, characterised by good corrosion resistance, was used as the skin, whereas AA7020, characterised by higher tensile strength, was used as the stringer. All welding experiments were performed with a constant tool tilt angle of 2° and under position control. FSW joints were produced using two tools with the same MX Triflute geometry, comprising a 19 mm diameter spiral shoulder and a threaded conical pin (diameters from 6.5 to 8.7 mm) with different lengths. The tools used in the study are shown in Figure 2; the top image presents the side view, and the bottom image presents the shoulder view. The pin of the first tool (tool 1) was 4.8 mm long (Figure 2a), whereas that of the second tool (tool 2) was 6.8 mm long (Figure 2b).

Table 3 presents the sample IDs and welding parameters used in this study. In the welding experiments, tool rotational speeds of 400 rpm and 700 rpm and traverse speeds ranging from 50 mm/min to 200 mm/min were used with both tools.

The obtained joints were cut perpendicular to the welding direction to investigate the formation and distribution mechanisms of weld defects. The cross-section of each sample was then examined using a confocal microscope. The samples were ground using SiC abrasive papers under water cooling and mechanically polished using diamond suspension for microscopic observations. For metallographic observations by optical microscopy, the samples were etched with Keller’s reagent (2 mL HF (40%), 3 mL HCl (36%), 5 mL HNO_3_ (63%), and 190 mL H_2_O) for 45 s. Microscopic examinations were subsequently carried out using an OLYMPUS LEXT OLS 4100 digital microscope (Olympus Corporation, Tokyo, Japan). To analyse macroscopic defects, cross-sections of the welds were first observed and photographed at relatively low magnification. Subsequently, each weld region was observed and photographed at higher magnification to examine the defects in more detail. After completing the microscopic examinations, microhardness measurements were carried out at mid-thickness in two perpendicular directions: along the skin and along the stringer.

A detailed microstructural assessment was performed using scanning electron microscopy on the joints exhibiting the most promising mechanical properties. The joints were electrolytically polished using a Struers LectroPol polisher (Struers ApS, Ballerup, Denmark) in a perchloric acid-based electrolyte (Struers A2) at 5 V, −5 °C, and for 10 s. Specimens were then analysed using a Tescan Lyra 3 FEG-SEM (TESCAN ORSAY HOLDING, a.s., Brno, Czech Republic) equipped with an Oxford Instruments Symmetry EBSD camera (Oxford Instruments Nanoanalysis, High Wycombe, Wielka Brytania) and an UltiMax-100 energy-dispersive spectroscopy (EDS) detector (Oxford Instruments Nanoanalysis, High Wycombe, Wielka Brytania). Prior to the analyses, three regions within each specimen were selected for detailed characterisation based on macrostructural observations. Microfractographic analyses focused on characteristic areas of the welded joint: the stir zone (SZ) and the thermo-mechanically affected zone (TMAZ). The first area was located in the centre of the SZ; the second was at the TMAZ/SZ interface on the retreating side; and the third was at the SZ/TMAZ interface at the bottom of the weld nugget. The obtained data were analysed using Aztec Crystal software (version 2.2), with an initial denoising procedure applied. The results were presented as inverse pole figure maps overlaid with band contrast, with grain boundaries marked in black.

**Figure 3:**
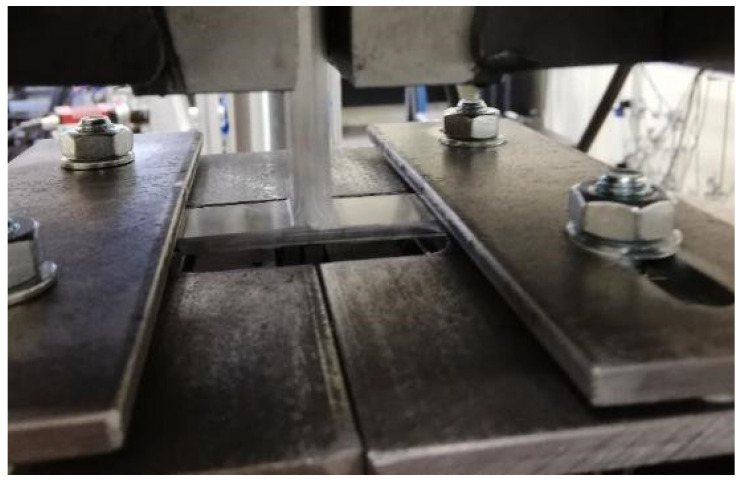
Specimen clamping arrangement in the dedicated fixture during the bending test.

Comparative bending tests were performed on at least three samples to evaluate joint strength and qualitatively compare dissimilar T-joints produced with different welding parameters and tool pin lengths. The tests were carried out using an Instron 8802 MTL testing machine (Instron, High Wycombe, UK). The T-joint specimens were tested using specially designed tooling mounted in the standard hydraulically actuated grips of the testing machine (Figure 3). The forces were measured using a load cell located in one of the specimen holders and then converted into bending moment values, which were plotted as a function of crosshead displacement.

## 3. Results and Discussion

### 3.1. Macrostructural Investigation

Macroscopic observations of the T-joints from the weld face produced using six different sets of welding parameters and two different tools did not reveal any defects that could disqualify the joints. Examination of the cross-sections of the weld regions for all specimens, presented in Table 4, provided more detailed information on the defects present in the FSW joints. The table shows the macrostructures of AA5083/AA7020 FSW T-joints as a function of the welding parameters for the two investigated Triflute pin lengths.

**Figure 4:**
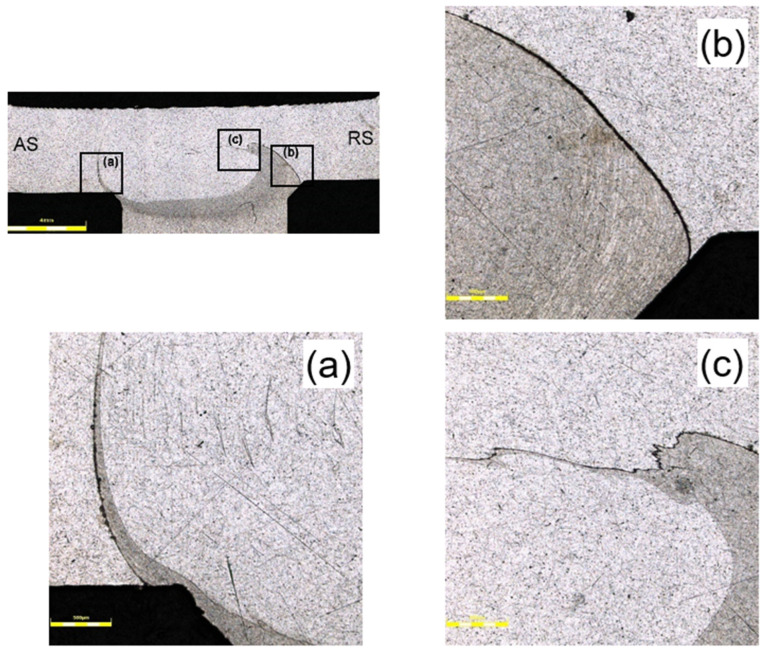
Examples of optical micrographs showing defects identified in the T-joint: (**a**) kissing bond, (**b**) hook defect, and (**c**) zigzag line.

**Figure 5:**
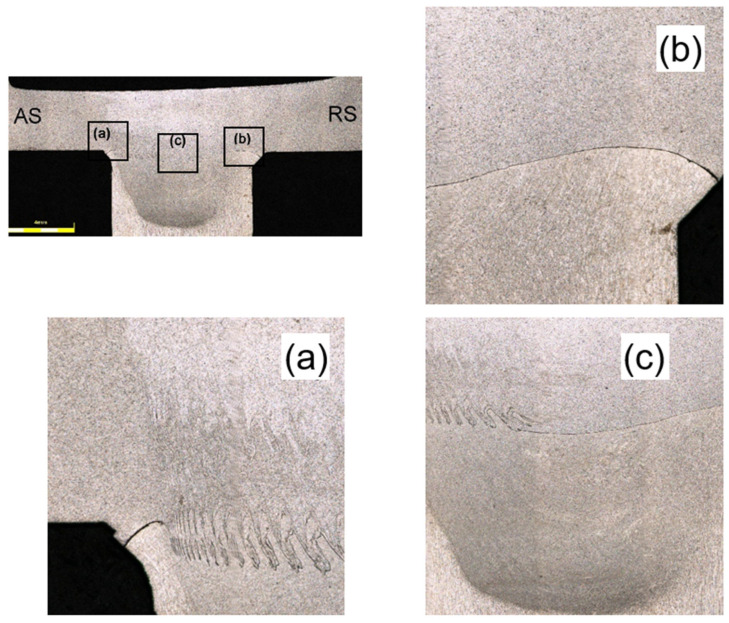
Example of optical microscopic observations of defects marked in the T-joint: OJLwSPD (**a**,**c**) hook defect (**b**), and zigzag line (**a**,**c**).

The left column of Table 4 shows joints produced using tool 1, whereas the right column shows joints produced using tool 2. Each row shows joints produced using the same welding parameters. The table also indicates the advancing side (AS) and retreating side (RS). In most macrographs, sound T-joints without apparent macroscopic defects and with smooth transitions between the skin and stringer can be observed. This indicates that most of the investigated combinations of FSW parameters are suitable for joining the dissimilar alloys in the T-joint configuration. The FSW parameters used in this study, listed in Table 3, therefore appear suitable for producing AA5083/AA7020 joints. However, some internal defects in the FSW T-joints require further analysis, as they may affect joint strength. The upper part of each macrograph corresponds to the AA5083 skin, whereas the vertical part corresponds to the AA7020 stringer. The advancing side (AS) and retreating side (RS) are marked in each macrograph. In the central part, the stir zone, where material mixing occurs, can be observed. The images in Table 4 show black, curved lines, which are not always considered defects. Some of the lines represent a boundary, visible only after etching, between the different aluminum alloys used to create the T-joint. Therefore, in the subsequent Figure 4 and Figure 5, characteristic features are identified in selected joints and described in detail.

The macrostructures of the joints presented in Table 4 reveal several defects associated with the FSW process when tool 1 was used (left column). The locations of the individual defects are indicated in Figure 4 and are discussed in detail below.

On the advancing side (AS) of the tool, a characteristic boundary between the two joined materials was observed in each case, extending vertically from the base of the stringer towards the weld face. On the retreating side (RS), this characteristic boundary followed a distinctly different path, extending from the base of the skin towards the stir zone. The visible defect in Figure 4a is referred to in the literature as a “kissing bond” [28]. This defect appears as a distinct and continuous boundary between the dissimilar materials, despite the absence of adequate bonding at the microstructural level. Several factors may contribute to the formation of this defect, including inappropriate welding parameters, differences in the mechanical and chemical properties of the joined materials, inadequate tool geometry resulting in insufficient material mixing, and the presence of an oxide layer on the material surfaces [29]. Such an oxide layer may act as a barrier between the materials and prevent complete bonding, even when no apparent defects are visible during external inspection of the joint.

Another common defect observed in the tested samples was the hook defect, shown in Figure 4b. This defect results from the deformation and upward bending of the material interface within the weld region, producing a characteristic hook-like shape. The hook defect adversely affects the mechanical strength of the joint and may reduce its overall quality and durability. Its formation is mainly influenced by inappropriate welding parameters, tool geometry, and the properties of the joined materials [30].

A characteristic line was also observed in some of the obtained T-joints, following an irregular path through the stir zone. This defect is referred to as a “zigzag line” and is visible in the weld cross-section (Figure 4c) as a continuous, irregularly shaped line. This line is considered to be a remnant of the oxide layer originally present on the material surfaces in FSW joints. Its formation may be associated with insufficient material stirring and disruption of the oxide layer, resulting from inadequate welding parameters or insufficient contact pressure between the joined materials [25,31].

In the case of T-joints produced using the second tool (tool 2), macroscopic observations (Table 4, right column) indicated an improvement in joint quality, reflected in enhanced material mixing and a reduced number of defects. For the investigated tool, the extent of the stir zone increased markedly towards the vertically oriented AA7020 stringer as a result of the greater pin length. However, changing the tool geometry only partially eliminated the defects observed when using the first tool. Microscopic analysis revealed a defect present in all of the obtained joints, known as the Original Joint Line with Severe Deformation (OJLwSD) [32]. This defect is shown in detail in Figure 5.

In FSW T-joints, the original interface between the two joined sheets undergoes severe plastic deformation during welding and may remain visible within the joint after the process [31]. This defect usually appears as a dark, curved line and can occur in various regions of the joint; however, it is most frequently observed on the retreating side and may also be interpreted as a hook defect, particularly at the interface between the joined materials (Figure 5b) [30]. The formation of the OJLwSPD defect in T-joints is attributed to three main factors. First, the action of the welding tool primarily promotes material displacement in the plane of tool rotation, while its effect on vertical material flow is limited, which may hinder upward material transport. Second, the spaces between the joined components must be filled by the flowing material, which may promote defect formation along the internal joint interfaces. Third, material flow during welding is asymmetric with respect to the joint axis, resulting in different deformation conditions on the advancing and retreating sides and promoting the formation of the OJLwSPD defect on the retreating side.

### 3.2. The EBSD and EDS Analyses

**Figure 6:**
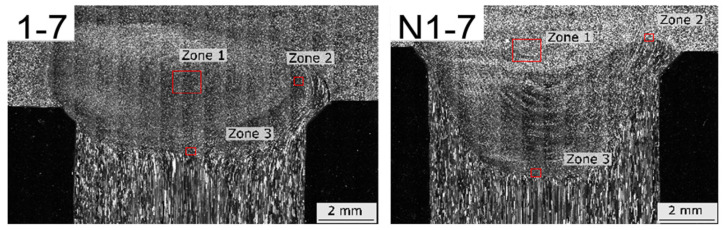
Macroscopic images of the joints 1-7 and N1-7, with marked areas for subsequent EBSD analysis.

**Figure 7:**
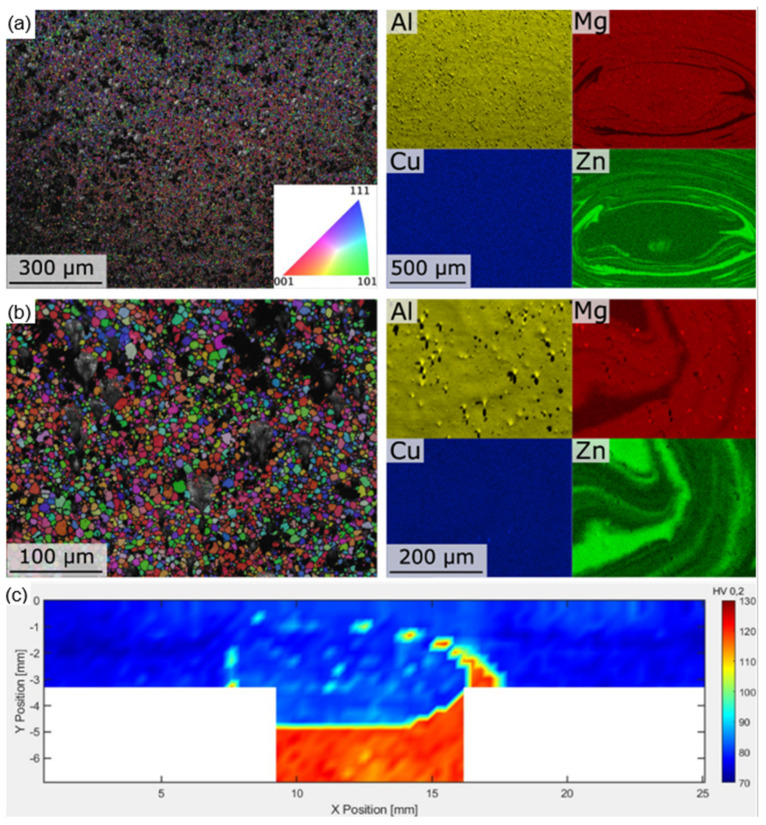
IPF map, microhardness distribution, and the corresponding maps of the principal elements distribution of the SZ of 1-7 specimens; (**a**) macroscopic and (**b**) detailed view, (**c**) microhardness distribution.

**Figure 8:**
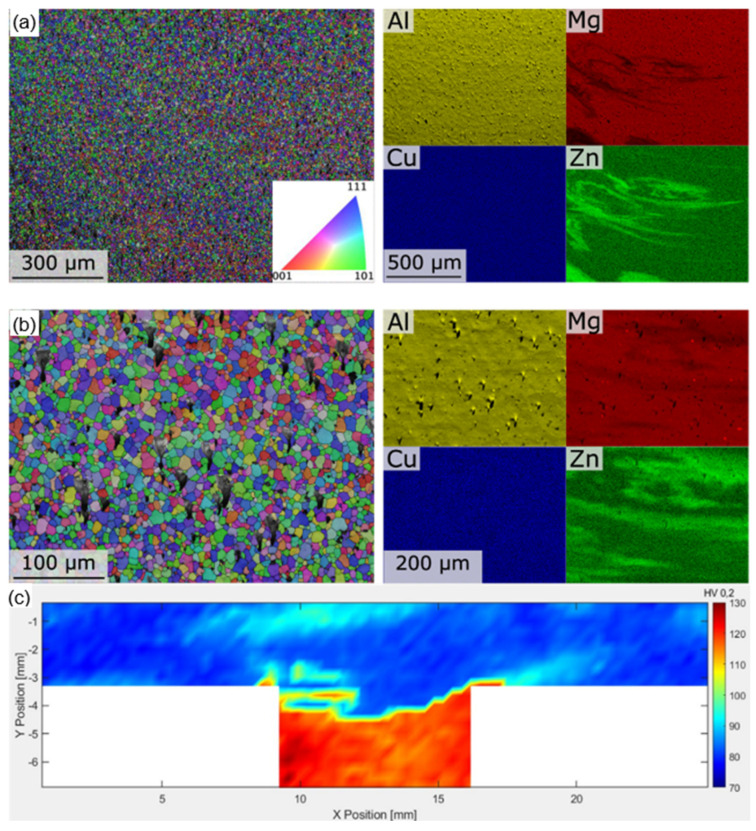
IPF map, microhardness distribution, and the corresponding maps of the principal elements distribution of the SZ of N1-7 specimen; (**a**) macroscopic and (**b**) detailed view, (**c**) microhardness distribution.

**Figure 9:**
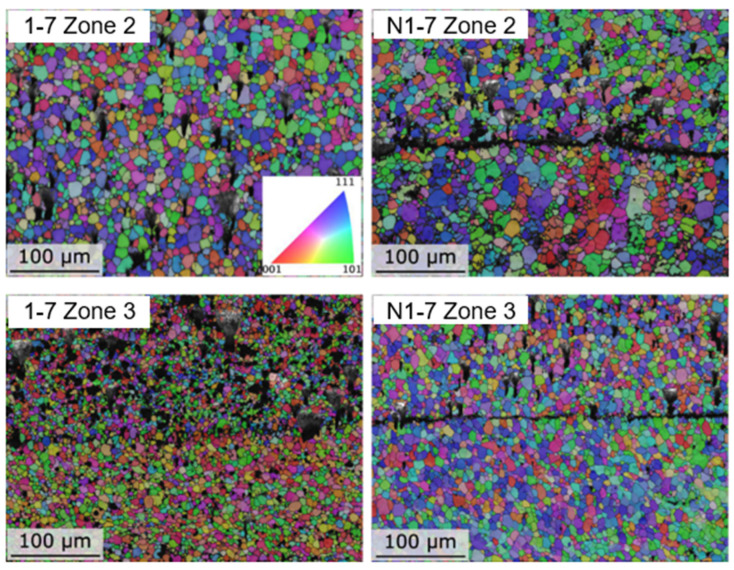
Comparison of the different zones of 1-7 and N1-7 joints, IPF maps.

The results of the EBSD and EDS analyses of the selected joints (1-7 and N1-7) are shown in Figure 6, Figure 7, Figure 8 and Figure 9. The macroscopic images in Figure 6 indicate the regions selected for detailed analysis. The different microstructural zones within the joints are clearly visible, revealing variations in stir-zone geometry resulting from the different tool dimensions.

The central part of the SZ in both joints (Figure 7 and Figure 8) shows a fine-grained structure with no pronounced preferential crystallographic orientation. The corresponding EDS maps clearly demonstrate the chemical intermixing of the dissimilar alloys, while the inverse pole figure (IPF) maps confirm the absence of apparent microstructural defects and indicate effective integration of the two dissimilar materials. The microhardness maps (Figure 7c and Figure 8c) reveal a clear correlation between local chemical composition and mechanical response. Regions enriched in Zn, corresponding to AA7020-derived material, exhibit locally elevated hardness values compared to the surrounding Mg-rich AA5083-derived matrix, confirming that elemental mixing directly governs the hardness distribution within the stir zone.

Zn-rich areas in both joints exhibited slightly larger grain sizes (Figure 7b and Figure 8b), most likely due to the much coarser, elongated grains of the AA7020 base material. Similar microstructures were also observed in the other zones. In the case of zone 2 (Figure 9), specimen N1-7 exhibits a clear interface between the SZ and the TMAZ of the AA7020 alloy.

The TMAZ side of the interface exhibits a coarser-grained structure, with groups of grains sharing similar crystallographic orientation, reflecting remnants of the original grain structure of the extruded product. A similar zone, analysed in the 1-7 specimen, lacked a clear interface, suggesting a greater degree of thermomechanical deformation. Zone 3 in both joints shows similar microstructural features, with a clear SZ/TMAZ interface in both cases and a coarser-grained structure within the TMAZ of the AA7020 alloy. However, due to the zone’s thermomechanical history, the original extruded grain structure cannot be recognized.

### 3.3. Microhardness Distribution

**Figure 10:**
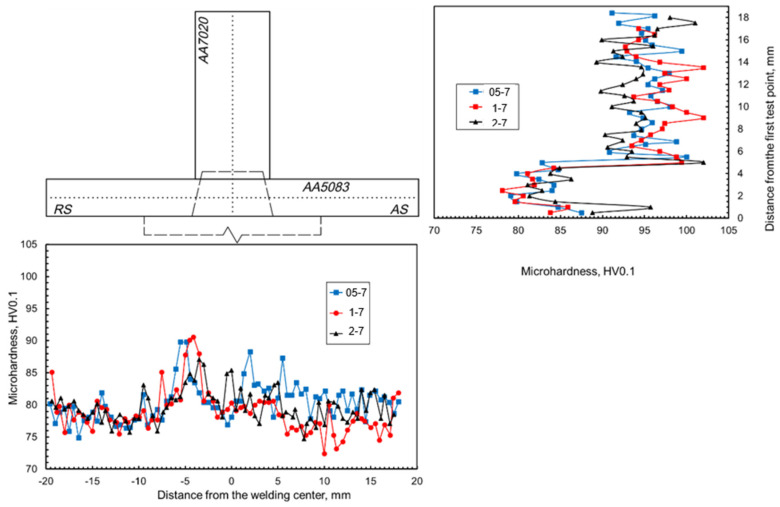
Microhardness profiles of samples 05-7, 1-7, and 2-7 produced using tool 1, measured along the stringer direction (**right-hand graph**) and the skin direction (**lower graph**).

**Figure 11:**
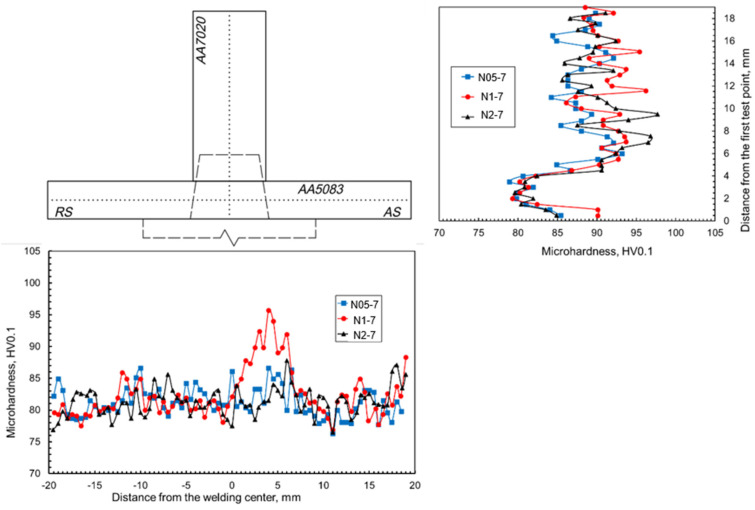
Microhardness profiles of samples N05-7, N1-7 and N2-7 produced using tool 2, measured along the stringer direction (**right-hand graph**) and the skin direction (**lower graph**).

The Vickers microhardness measurements across the T-joint cross-sections were performed along two directions corresponding to the orientations of the skin and stringer, as shown in Figure 10 and Figure 11. The tool profile is marked with a dashed line in both figures. The microhardness profiles were grouped for the same tool rotational speed and geometry but different traverse speeds. According to our previous study [27], the base-material hardness values were 86 HV0.1 and 103 HV0.1 for AA5083-H111 and AA7020-T651, respectively. Along the profiles measured in the skin direction (AA5083), no significant changes in base material hardness were observed within the heat-affected zones for either tool. Only a slight increase in hardness was observed in the vicinity of the stringer on the retreating side for tool 1, which may indicate limited mixing of the stringer material into this region of the joint.

For microhardness profiles measured along the stringer direction, the microhardness values showed considerably greater variation. This was mainly due to the transition from the AA5083 skin, with a microhardness of 85 HV0.1, to the AA7020 stringer, with an average microhardness of 98 HV0.1. A typical feature was a significantly higher microhardness value at the first or second measurement point, which can be attributed to local grain refinement in the AA5083 portion of the stir zone, similar to the observations reported in [15,16]. Since AA7020 is a precipitation-hardenable alloy, the heat generated during FSW generally leads to relatively pronounced reductions in strength in this group of materials, manifested, among other effects, by a decrease in microhardness [33]. However, in the present study, no extensive softened region within the heat-affected zone was observed in the AA7020 stringer. This behaviour can be attributed to the limited amount of heat introduced into the AA7020 alloy by the rotating tool. Considering that the heat generated by friction between the tool and the workpiece is distributed as follows: 86% at the shoulder, 11% at the pin side surface, and 3% at the pin tip [34], in the case of the tool with a 4.8 mm-long pin (Figure 10), only the pin tip and a very small portion of the pin side surface transferred heat directly into the AA7020 stringer. Although the short pin limited direct frictional heating of the stringer, heat still reached it by conduction from the shoulder-heated skin, which explains why the reduced-hardness zone in the AA7020 is shallow (about 4–5 mm). The hardness recorded in the heat-affected zone of the AA7020 alloy (approximately 80–85 HV0.1) is consistent with literature data [35] and, as in other precipitation-hardened aluminium alloys, is attributed to the dissolution of strengthening precipitates. Despite the substantial grain refinement in the stir zone (Figure 9), grain-boundary strengthening is insufficient to compensate for the loss of strength associated with dissolution and overageing of the strengthening phase [36].

An additional factor limiting the interpretation of the microhardness reduction within the AA7020 portion of the stir zone is the partial intermixing with the AA5083 alloy, which prevents the measured microhardness values from being attributed to a single specific material. Phenomena associated with potential grain growth in the HAZ (Figure 6) can be considered of secondary importance compared with the strength reduction caused by adverse thermally induced transformations of the strengthening phase [33]. Interestingly, increasing the pin length did not significantly affect the microhardness distribution in the AA7020 alloy. A softened zone was still observed at a depth of approximately 4–5 mm, with a hardness of about 85 HV0.1, while the region between 6 and 8 mm exhibited a relatively uniform hardness of approximately 92 HV0.1, corresponding to the location of the stir zone. The absence of any shift in the softened zone with increasing pin length supports the hypothesis that an intermediate layer formed between the two alloys as a result of their intermixing, extending to a depth of approximately 1 mm into the AA7020 stringer.

### 3.4. Strength of T-Lap Joints

**Figure 12:**
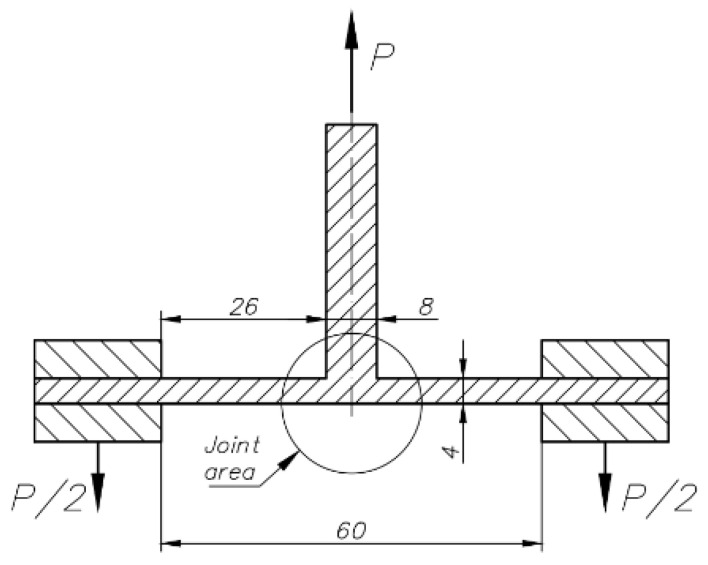
Schematic diagram of the T-joint bending test.

Comparative bending tests were performed under pull-out loading to evaluate joint strength and qualitatively compare the dissimilar T-joints. T-joints are commonly used in aircraft, shipbuilding, and railway structures. In these structures, joints are rarely subjected to pure axial tension. They are most often subjected to asymmetric bending, lateral loads, and vibrations, which generate high bending moments in the transition zone. Bending testing directly simulates the most critical stress states. The FSW process in the T-configuration carries the risk of specific weld imperfections, such as kissing bonds, hooking defects, or tunnelling defects. In a tensile test, these flaws (especially hooking or kissing bonds on the retreating side) may not be fully activated because of the direction of force application. In turn, the bending test generates maximum tensile stresses in the outer fibres of the weld, which immediately initiates cracking at the locations of these micro-defects [37]. The T-joint specimens were tested using specially designed tooling mounted in the standard hydraulically actuated grips of the testing machine (Figure 3). The bending-load configuration used during the tests is shown in Figure 12.

The FSW T-joints were loaded with a force P, as shown in Figure 12, and supported by the clamps at a distance of approximately 26 mm from the joint region. The joint was secured using bolted connections. Based on the loading configuration and the adopted simplifications, the bending moment was determined using Equation (1)

Mg = (P⋅l)/4 [Nm](1)

where

P—force acting on the tested sample during the bending test [N];
l—force lever arm [m].

**Figure 13:**
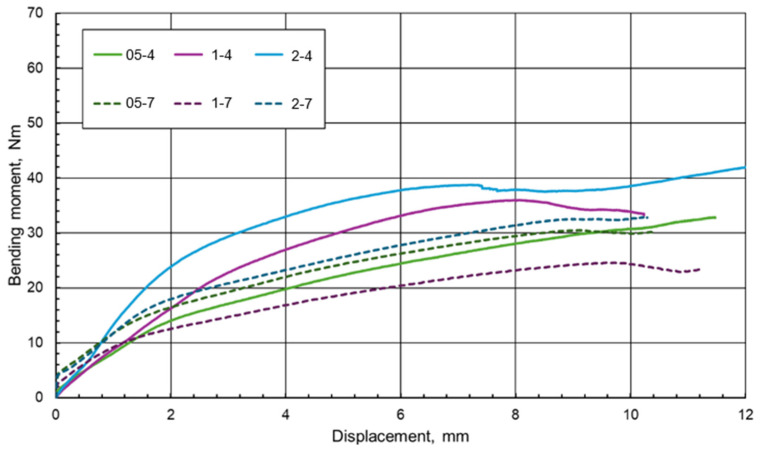
Bending moment–displacement curves for specimens produced using tool 1 at two rotational speeds of 400 rpm (solid lines) and 700 rpm (dashed lines), and at traverse speeds of 50 mm/min, 100 mm/min and 200 mm/min.

**Figure 14:**
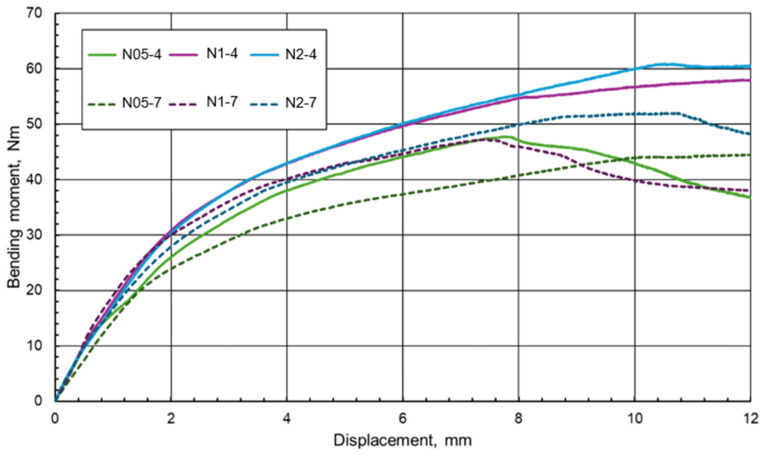
Bending moment–displacement curves of specimens produced using tool 2 at two rotational speeds of 400 rpm (solid lines) and 700 rpm (dashed lines), and at traverse speeds of 50 mm/min, 100 mm/min and 200 mm/min.

The displacement of the specimens was measured until the machine crosshead reached a maximum relative displacement of 12 mm or until the test specimen fractured. The strength test results were used to generate bending moment-displacement curves that summarise the effect of specific joining parameters on T-joint strength. This comparison allows a more reliable assessment of joint quality, depending on the tool used and the welding parameters. The curves obtained for the pull-out bending tests at a constant tool rotational speed and different traverse speeds are presented in Figure 13 and Figure 14, respectively. 

Based on the observations in Figure 13 for tool 1, it can be concluded that only one specimen, produced at a rotational speed of 400 rpm and a traverse speed of 200 mm/min, achieved the target displacement of 12 mm. In contrast, for the other welding parameter combinations, the peak bending moment was reached and joint failure occurred earlier.

**Figure 15:**
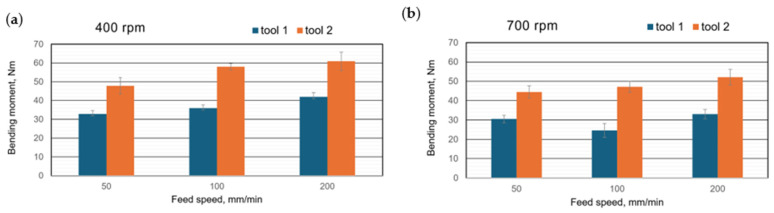
Effect of welding parameters and pin length on joint strength at rotational speeds of: (**a**) 400 rpm and (**b**) 700 rpm.

Changing the tool geometry (longer pin) while maintaining the previously adopted FSW process parameters significantly affected the bending test results (Figure 14), increasing the bending moment required to achieve a displacement comparable to that obtained with tool 1. Each tested specimen achieved the previously assumed displacement value of 12 mm. The specimens tested at a constant traverse speed of 200 mm/min and different rotational speeds demonstrated the highest joint strength. Based on the bending moment curves presented in Figure 14, increasing the rotational speed from 400 to 700 rpm resulted in approximately 15% lower joint strength for a given traverse speed. Figure 15 presents the test results for two tool rotational speeds: 400 rpm (Figure 15a) and 700 rpm (Figure 15b), three traverse speeds (50 mm/min, 100 mm/min and 200 mm/min) and two tools with different pin lengths (tool 1 and tool 2).

At a rotational speed of 400 rpm (Figure 15a), a clear and systematic increase in joint strength was observed with increasing traverse speed from 50 mm/min to 200 mm/min, regardless of the tool used. Comparison of the results for both tools indicates that, under all analysed conditions, tool 2 provided significantly higher joint strength than tool 1. These differences were particularly pronounced for higher traverse speeds, where tool 2 provided joint strengths several tens of percent higher. This phenomenon may indicate that the longer pin of tool 2 promoted more intensive material mixing in the stir zone and better filling of the T-joint corner region. The relatively low rotational speed, combined with the higher traverse speed, helped to maintain stable thermomechanical conditions during the process. Limiting excessive heat input could prevent local overheating of the material, thereby promoting a favourable microstructure in the joint zone and, consequently, higher joint strength. Increasing the tool rotational speed to 700 rpm (Figure 15b) reduced joint strength across the entire traverse-speed range, which is consistent with the results reported by Di Bella et al. [17]. As with 400 rpm, a trend of increasing strength with increasing traverse speed was observed, but these values were significantly lower. At this rotational speed, tool 2 also demonstrated an advantage over tool 1, confirming the significant role of pin length in shaping the mechanical properties of FSW joints. Particularly unfavourable results were obtained with tool 1 at a traverse speed of 100 mm/min, which may indicate an imbalance in the thermal conditions of the process, leading to excessive material plasticization and weakening of the joint structure. Higher rotational speeds result in increased heat input to the joint, which, in the case of T-joints and alloys with different thermal conductivities and strengthening properties, can lead to microstructural degradation, grain growth, or inhomogeneity in the stir zone.

Table 5 illustrates examples of samples that exhibited visible crack zones following bending tests conducted on friction stir welded (FSW) AA5083/AA7020 T-joints. The table includes photographs of the samples arranged in groups of three, all produced under identical rotational speeds and utilizing the same welding tool. Observations from cracked samples, particularly at a pin length of 4.8 mm, revealed repetitive cracking mechanisms in the T-joints, primarily associated with hook and kissing-bond defects. The initiation of cracks predominantly occurred on the retreating side (RS) of the joint and displayed a tendency to propagate toward the centre of the weld (see Table 5, Tool 1). Additionally, kissing bond defects contributed to crack initiation at the corners of the joint. These defects were identified during macroscopic evaluations (refer to Table 4) and are often present outside the stir zone, where the materials come into contact without establishing a chemical or mechanical bond. The occurrence of such defects resulted in diminished strength properties during the testing of T-joints compared to those produced with Tool 2, as evidenced by the results presented in Figure 15.

The 6.8 mm pin-length tool exhibited repetitive fracture mechanisms in T-joints during friction stir welding that were significantly different from those observed for Tool 1. The primary fracture mechanism identified involved damage to the oxide layer at the primary contact surface between the plates, resulting in the separation of the stiffener from the plate (refer to Table 5, Tool 2). Within these T-joints, crack initiation frequently occurred at the corner fillets, where defective material mixing was evident, subsequently propagating along the interface between the joined materials. Enhanced oxygen concentrations, previously reported in energy-dispersive spectroscopy (EDS) studies, were detected along the fracture line, suggesting the presence of a continuous oxide film that impeded the diffusion and fusion of the materials. Notably, the fracture mechanism observed during bending tests proved to be advantageous, as indicated by the superior strength of the T-joints, as illustrated in Figure 15.

## 4. Conclusions

Based on the conducted experiments and analysis of dissimilar AA5083/AA7020 friction stir welded T-joints, the following conclusions can be drawn.

- Sound dissimilar T-joints can be produced by friction stir welding without visible surface defects; however, internal imperfections such as hook defects, kissing bonds, and original joint lines with severe plastic deformation can be seen on micrographs. This means that, in this work, it was not possible to obtain fully satisfactory T-shaped joints by eliminating these defects entirely. Still, our observations show that their influence on joint strength is strongly dependent on pin length and process parameters.
- Increasing the traverse speed from 50 mm/min to 200 mm/min significantly improves joint bending strength, particularly when combined with a lower rotational speed. For the tool with a 4.8 mm-long pin, the increase was approximately 12%, and for the tool with a 6.8 mm-long pin, it exceeded 20% at a rotational speed of 400 rpm.
- A rotational speed of 400 rpm provides a more favourable thermomechanical balance than 700 rpm, resulting in higher joint strength and improved structural integrity.
- The use of a tool with a longer pin (6.8 mm) enhances material flow in the joint region, reduces defect severity, and leads to a noticeable improvement in mechanical performance. The observed increase in the average bending moment value was at least 28% for each set of FSW parameters.
- For the investigated material configuration and tool geometries, the most advantageous combination of welding parameters was identified as a rotational speed of 400 rpm, a traverse speed of 200 mm/min, and the tool with the longer pin.

The results demonstrate the effectiveness of friction stir welding for producing high-strength dissimilar aluminium alloy T-joints and highlight the importance of pin length and heat input control in advanced lightweight structural applications.

## Figures and Tables

**Table 1:** Chemical composition of base aluminium alloys used in the research (wt. %) [27].

	Al	Mg	Mn	Si	Fe	Cr	Zn	Ti	Cu
**AA5083**	Bal.	**4.34**	0.63	0.076	0.13	0.064	0.035	0.055	0.032
**AA7020**	Bal.	**1.30**	0.24	0.16	0.32	0.14	**4.7**	0.034	0.05

**Table 2:** Mechanical properties of basic materials according to our own research [27].

	Tensile Strength (MPa)	0.2% Yield strength (MPa)	Elongation (%)	Microhardness HV0.1
**AA5083-H111**	310	165	20.2	82
**AA7020-T651**	390	300	8.6	107

**Table 3:** Specimen IDs and welding parameters used in the study.

Sample ID (Tool 1)	Sample ID (Tool 2)	Traverse Speed (mm/min)	Rotation Speed (rpm)
**05** **-** **4**	N05-4	50	400
**1** **-** **4**	N1-4	100	400
**2** **-** **4**	N2-4	200	400
**05** **-** **7**	N05-7	50	700
**1** **-** **7**	N1-7	100	700
**2** **-** **7**	N2-7	200	700

**Table 4:** Macrostructures of AA5083/AA7020 FSW T-joints produced using different welding parameters.

Sample ID	Tool 1	Tool 2
**0.5-4** **N0.5-4**	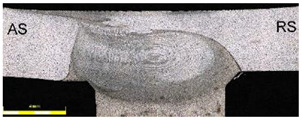	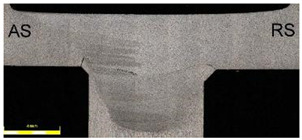
**1-4** **N1-4**	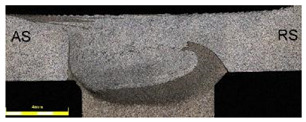	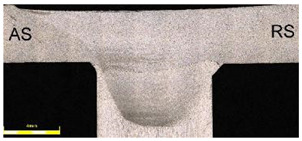
**2-4** **N2-4**	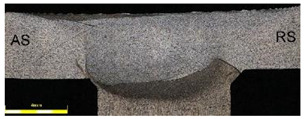	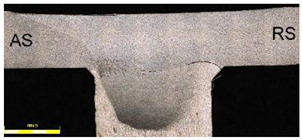
**05-7** **N05-7**	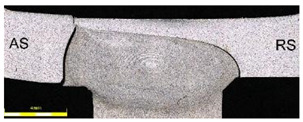	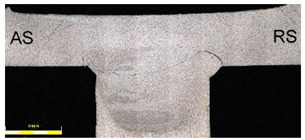
**1-7** **N1-7**	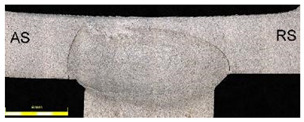	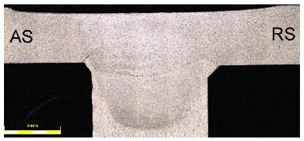
**2-7** **N2-7**	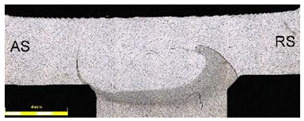	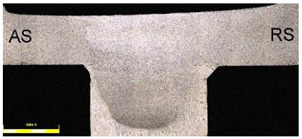

**Table 5:** Examples of samples after bending tests with visible crack zones in the FSW AA5083/AA7020 T-joints.

Sample	Tool 1 (4.8 mm Pin Length)
**05-4** **1-4** **2-4**	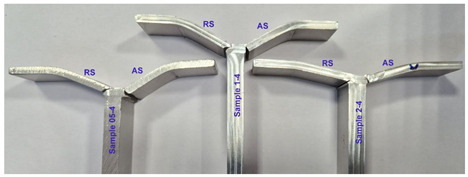
**05-7** **1-7** **2-7**	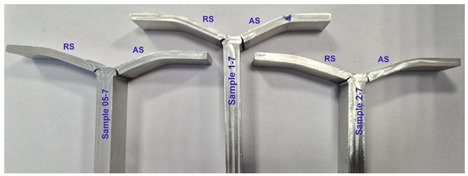
	**Tool 2 (6.8 mm pin length)**
**N05-4** **N1-4** **N2-4**	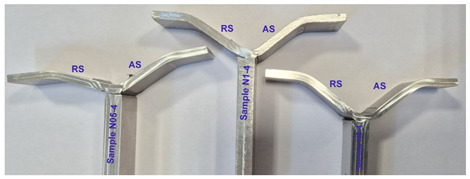
**N05-7** **N1-7** **N2-7**	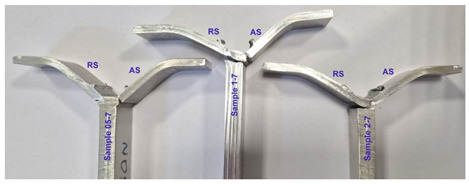

## Data Availability

The original contributions presented in this study are included in the article. Further inquiries can be directed to the corresponding author.
